# Comprehensive analysis of mutational and clinicopathologic characteristics of poorly differentiated colorectal neuroendocrine carcinomas

**DOI:** 10.1038/s41598-021-85593-9

**Published:** 2021-03-18

**Authors:** Sun Mi Lee, Chang Ohk Sung

**Affiliations:** 1 Department of Pathology, Jeju National University Hospital, 15 Aran 13-gil, Jeju-si, 63241 Jeju-do South Korea; 2 Department of Pathology and Molecular Diagnostic Laboratory, Asan Medical Center, University of Ulsan College of Medicine, Seoul, South Korea

**Keywords:** Cancer genomics, Colorectal cancer

## Abstract

Poorly differentiated neuroendocrine carcinoma (NEC) is a rare subtype of colorectal cancer (CRC). This study aimed to investigate clinicopathologic characteristics of colorectal NECs and elucidate genomic differences and similarities between colorectal NECs and colorectal adenocarcinomas (ACs). A total of 30 colorectal NECs were screened for frequently identified CRC oncogenic driver genes by targeted next-generation sequencing of 382 genes. The median age of the patients was 67 years (range, 44 to 88 years). NECs occurred predominantly in the rectum (47%) and exhibited multiple adverse prognostic pathologic factors, including frequent lymphatic and vascular invasions, high rates of lymph node metastasis and distant metastasis and advanced TNM stage. The 1-, 3-, and 5-year overall survival rates of NEC patients were 46.7%, 36.4%, and 32.7%, respectively, with a median overall survival period of 11.5 months. In a molecular analysis, NECs showed high rates of *BRAF* mutation (23%), predominantly p.V600E (71%), and alterations in *RB1* (47%), particularly deletion (57%). The frequencies and distributions of other genes, such as *KRAS*, *APC*, *SMAD4*, and *PIK3CA*, and microsatellite instability status were similar to those of ACs. These findings provide beneficial information for selecting therapeutic options, including targeted therapy, and a better understanding of the histogenesis of this tumour.

## Introduction

Poorly differentiated neuroendocrine carcinoma (NEC) in the colorectum is rare, accounting for approximately 0.6% of all colorectal carcinomas (CRCs)^1,2^. The majority of patients present metastatic disease at the time of diagnosis^3^. Colorectal NECs exhibit aggressive biologic behaviour with short-lived responses to therapy and inferior prognosis compared with typical CRCs^4,5^. Treatment strategies for colorectal NECs generally follow the chemotherapy regimens used for pulmonary NECs, consisting of platinum-based chemotherapy as the first-line therapy. Nonetheless, the prognosis of colorectal NECs remains dismal, with a low median survival of 5–11 months^1^.

According to the World Health Organization (WHO) classification of gastrointestinal neuroendocrine tumours, NECs are characterized by high-grade morphology, frequent necrosis, increased mitotic figures (> 20 per 2 mm^2^), and a high Ki-67 index (> 20%)^6^. Morphologically, NECs are divided into small-cell or large-cell types, similar to their pulmonary counterparts. Recently, it has been recognized that there are a subset of well-differentiated neuroendocrine tumours with a high proliferation index (a Ki-67 index > 20% and a mitotic rate usually < 20 per 2 mm^2^)^5^. Although well-differentiated neuroendocrine tumours with a high proliferation index are classified as “grade 3 tumours” according to the current WHO classification system, they show different molecular alterations and decreased response to platinum-based therapy compared with small-cell or large-cell NECs^5^. Thus, poorly differentiated NEC is considered a different disease entity that is distinct from well-differentiated neuroendocrine tumours with a high proliferation index, WHO grade 3.

Several prior studies have investigated the genetics of colorectal NECs which have been reported to have frequent alterations in *RB1* as well as in genes commonly identified in colorectal adenocarcinomas (ACs) including *TP53*, *APC*, *KRAS*, *PIK3CA*, *BRAF* etc^7–10^. *BRAF* mutation frequencies have been reported variably, ranging from 4 to 59%, as revealed by Sanger sequencing or targeted next-generation sequencing. In addition, *KRAS* mutations occur in 8% to 70% of cases, and the frequency of *TP53* mutation reportedly ranges from 21 to 80%. In a recent series of 25 colorectal NECs, Shamir et al. found *RB1* alterations in 14 tumours (56%), with *TP53* being mutated in 12 (48%), similar to the frequencies of small-cell carcinoma of the lung^11^. In contrast, other frequently identified somatic mutations in colorectal ACs, such as mutations in *BRAF*, *KRAS*, and *PIK3CA,* but not in *APC,* were less common than in other studies^7–10^. Due to the rarity of this disease entity, the frequencies of identified mutations in colorectal NECs have been inconsistently described. Therefore, our study adds an additional 30 pure NEC cases to the previous literature, which helps to validate the prior molecular data of colorectal NECs.

It is known that different frequencies and distributions of mutations in key oncogenes and tumour suppressors exist between right- and left-sided CRCs. In comparison analysis of frequently identified somatic mutations in right- and left-sided CRCs from the Cancer Genome Atlas dataset, *BRAF* mutations, particularly p.V600E, are significantly more common in right-sided CRC (24.2% vs. 2.1%)^12^. Right-sided CRCs had higher rates of microsatellite instability and *PIK3CA* mutations and increased mutational burden, whereas mutations in *APC* and *TP53* were enriched in left-sided CRC (81.9% vs. 63.6% and 64.6% vs. 34.8%). Considering that the different mutational rates of key oncogenic driver genes of CRCs depend on the tumour site, a specific analysis would involve comparison of identified mutations of colorectal NECs with those of colorectal ACs after tumour site matching.

The main purpose of this study was to better elucidate the clinicopathologic features and clinical outcome of colorectal NECs and genomic distinctions as well as similarities between these tumours and conventional ACs. We also investigated potential therapeutically targetable molecular alterations in colorectal NECs to optimize patient selection for new drugs and their combinations.

## Methods

### Patient selection and histopathology

The institutional review board of Asan Medical Center (AMC), Seoul, South Korea, approved this study. All methods were performed in accordance with relevant guidelines and regulations. We reviewed the records of patients diagnosed with poorly differentiated NECs and neuroendocrine tumours of WHO grade 3 in the colorectum from the Pathology Department at AMC. A total of thirty-five surgically resected cases were initially searched and reviewed. Neuroendocrine markers including synaptophysin and chromogranin and Ki-67 staining were performed for all thirty-five cases. Among them, well-differentiated neuroendocrine tumors of grade 3 (Ki-67 > 20% or mitoses > 20/2 mm^2^) and posttherapy specimens of colorectal NECs were excluded. Mixed adenoneuroendocrine carcinoma (MANEC) is a mixed malignant neoplasm with a neuroendocrine component combined with a glandular component. Each component should account for at least 30% of the tumor cell population^6^. To maximize DNA extraction of the pure component of NEC for molecular analysis, cases of MANEC were also excluded. Poorly differentiated NEC cases with more than 70% neuroendocrine components were selected for molecular and immunohistochemical analyses. Tumours were morphologically classified into small-cell or large-cell types according to the WHO classification criteria^6^. Tumours consisting of small to medium-sized cells with a high nuclear-cytoplasmic ratio, scant cytoplasm, and fusiform nuclei containing fine granular chromatin without prominent nucleoli were considered small-cell NEC. Large-cell NECs were considered those consisting of large polygonal cells with round nuclei, moderate amounts of cytoplasm, and sometimes prominent nucleoli. The mitotic index was obtained by evaluating the most mitotically active 2 mm^2^ area of the tumour. For a control group, initially, 120 surgical cases of colorectal AC after matching the tumour site were searched from the pathology report profile and reviewed. In cases of suspicious neuroendocrine differentiation within a tumour, immunohistochemistry for neuroendocrine markers was performed to confirm neuroendocrine differentiation. Ultimately, 100 surgical cases of colorectal ACs without neuroendocrine differentiation were selected as a control group. Histologic findings including mitotic count, depth of invasion, lymphatic invasion, vascular invasion, perineural invasion, regional lymph node metastasis, distant metastasis, and pathologic TNM staging were also evaluated. Tumours were staged according to the 8^th^ edition of the American Joint Committee on Cancer (AJCC) TNM staging system for CRCs after the clinicopathologic features and radiologic findings had been re-reviewed^13^. Demographic and clinical information, including age, sex, underlying disease, type of surgery, tumour site, date of diagnosis, postoperative treatment, last follow-up status, date of death or last follow-up, were collected from a patient medical record review.

### Immunohistochemistry for p53, Rb1, p16, and mismatch repair proteins

Immunohistochemistry was performed on representative whole tissue sections using the avidin–biotin method. The primary antibodies used were against MLH1 (mouse monoclonal antibody clone G168-728 at a dilution of 1:300; Cell Marque, CA, USA), MSH2 (mouse monoclonal antibody clone FE11 at a dilution of 1:100; Calbiochem, CA, USA), MSH6 (mouse monoclonal antibody 44 at a dilution of 1:300; BD Transduction Laboratories, CA, USA), PMS2 (mouse monoclonal antibody clone A16-4 at a dilution of 1:125; BD Transduction Laboratories, CA, USA), p53 (mouse monoclonal antibody clone DO-7 at a dilution of 1:1,500; DAKO, Glostrup, Denmark), Rb1 (clone 3C8 at a dilution of 1:10,000; QED Bioscience, CA, USA), p16 (clone E6H4; prediluted; Ventana, AZ, USA), anti-Human papillomavirus (HPV, mouse monoclonal antibody clone K1H8 at a dilution of 1: 400; DAKO, Glostrup, Denmark). Anti-HPV is immunoreactive with paraffin sections of formalin-fixed tissues infected with HPV type 6, 11, 16, 18, 31, 33, 42, 51, 52, 56 and 58. An automated stainer (Ventana Medical Systems, AZ, USA) was used according to the manufacturer’s protocol. Immunohistochemical expression of each MMR protein was considered intact if nuclear staining of neoplastic cells was detected with internal control positivity in the non-neoplastic crypt epithelium. Expression of p53 was considered aberrant if there was diffuse and strong nuclear staining (more than 2/3 of cells) or complete loss of expression. Rb1, p16, and HPV were classified as immunoreactive if nuclear staining (Rb1 and HPV) or cytoplasmic staining (p16) in all neoplastic cells was observed. After reviewed p16 staining of colorectal NEC tumours, immunohistochemistry for HPV was performed on all p16 positive tumours to detect HPV infection.

### Microsatellite instability testing

Microsatellite instability (MSI) analysis was performed on colorectal NEC tumours by multiplex polymerase chain reaction (PCR) with five quasimonomorphic mononucleotide repeat markers: BAT25, BAT26, D5S346, D17S250 and D2S123. Genomic DNA was isolated from paraffin-embedded tumour tissues using a QIAamp DNA Mini Kit (Qiagen, CA, USA). Each primer was end-labelled with one of the following fluorescent markers: FAM, HEX, or NED. An ABI Prism 3130 Genetic Analyzer (Applied Biosystems, CA, USA) was used to analyse the products, and allelic sizes were estimated by Genemapper 4.1 (Applied Biosystems, CA, USA). Tumours with allelic size variation in two or more of the microsatellite markers were deemed to be MSI-high, whereas tumours with allelic variations in one of the microsatellites were classified as MSI-low. If there were no allelic size variations, all microsatellites were considered microsatellite-stable (MSS).

### Targeted next-generation sequencing of a 382-gene panel

After a review of matched haematoxylin and eosin-stained slides, formalin-fixed and paraffin-embedded (FFPE) tissue blocks containing adequate tumour cellularity (> 70%) were selected. The area with a pure neuroendocrine component of NEC was carefully circled by a molecular pathologist (C.S.). Two to five sections (6 µm thick) from the circled area in each FFPE tissue were obtained. After deparaffinization with xylene and ethanol, gDNA was isolated using NEXprep FFPE Tissue Kit (#NexK-9000; Geneslabs, Seongnam, Korea). Quantification was performed using Qubit dsDNA HS Assay Kit (Thermo Fisher Scientific, Waltham, MA, USA). Targeted next-generation sequencing was performed using MiSeq (Illumina, Inc., San Diego, CA, USA) with the OncoPanel AMC version 3.0 panel (OP-AMCv3, developed in-house by ASAN-CCGD). This panel covers approximately 1.2 Mb with 33,524 probes targeting a total of 382 genes, including the entire exons of 199 genes, 184 hot spots, and partial introns for eight genes often rearranged in cancer, as previously described^14^. Briefly, the cancer-related genes evaluated included *ABL1, AKT1, AKT2, ALK, APC, ARID1A, BRAF, CDH1, CSF1R, CTNNB1, CDKN1A, CDKN1B, CDKN2A, CDKN2B, CDKN2C, EGFR, ERBB2, ERBB3, ERBB4, FBXW7, GNAS, HRAS, NRAS, PIK3CA, PIK3CB, PIK3CD,* and *SMAD4,* among others. Overall, the panel covered 823,971 bp. A DNA library was prepared as described in our previous report using the S1 method^14^. Each library was constructed with sample-specific barcodes six-bp in size and quantified using Qubit Kit. Eight libraries were pooled for hybrid capture using the Agilent SureSelect XT custom kit (OP-AMCv3RNA bait, 1.2 Mb; Agilent Technologies, CA, USA). The enriched target concentration was measured by quantitative polymerase chain reaction (qPCR; Kapa Biosystem, Inc., MA, USA). DNS libraries that passed quality checks were sequenced using MiSeq. Sequenced reads were mapped to the human reference genome (NCBI build 37) using Burrows-Wheeler Aligner (version 0.5.9) with default options. PCR duplicates were removed using the Picard tool. Then, de-duplicated reads were realigned at known indel positions using GATK IndelRealigner, and base quality was recalibrated with GATK Table Recalibration^15^. Somatic mutations of single-nucleotide variants and short indels were called in tumour tissue with matched normal tissue using MuTect (1.1.7) and SomaticIndelocator in GATK^15–17^. Germline variants from somatic variant candidates were filtered out using the common dbsnp database (build 141; found in ≥ 1% of samples), the Korean Reference Genome database^18^ and an in-house panel of normal variants^19,20^. Filtered somatic variants were annotated with Variant Effect Predictor (v79) and then converted to MAF files using vcf2maf (v1.612)^21^. False-positive variants were manually curated using Integrative Genomic Viewer^22^.

### Statistical analysis

Pearson's chi-square test or Fisher's exact test was applied to evaluate correlations between clinicopathologic variables and frequently mutated genes. Continuous variables were analysed using Student’s *t*-test or the Mann–Whitney *U*-test. Overall survival curves were constructed using the Kaplan–Meier method and compared with the log-rank test. Cox proportional hazard models were employed to estimate the combined influence of clinicopathologic variables on survival. A *p*-value < 0.05 was considered statistically significant.

### Ethics approval and consent to participate

Informed consent was obtained from all study participants. This study was approved by the institutional review boards at Asan Medical Center, Seoul, South Korea. The study was conducted in accordance with the Declaration of Helsinki.

## Results

### Clinicopathologic features of colorectal NECs and ACs

A comparison analysis of the clinicopathologic features of colorectal NECs and ACs is summarized in Table 1. For colorectal NECs, the patients consisted of twenty men and ten women (male to female ratio: 2:1), with a mean age of 67 years (range: 44 to 83 years). One patient each had a history of Lynch syndrome and familial adenomatous polyposis. The most common primary tumour sites were the rectum (47%), right-sided colon (30%), and left-sided colon (23%). The median tumour size was 5.65 cm (range, 1.6–16 cm). Large-cell morphology was more commonly observed than small-cell morphology (60% vs. 40%). The median mitotic count per 2mm^2^ was 62.5, ranging from 12 to 141/2mm^2^. The proliferation index assessed by Ki-67 was high, with a median percentage of 75% (range, 50%-95%). The coexisting component within NEC tumours was predominantly adenocarcinoma (23%) or tubular adenoma (10%). Metastatic disease was noted in 11 (36.7%) patients at diagnosis, with the liver being the most common organ involved (54.5%), followed by distant lymph nodes (27.3%) and the ovary or peritoneum (18.2%). In the comparison analysis of colorectal NECs and ACs, patients with colorectal NECs were found to be slightly older than those with AC (mean age: 67 years vs. 62 years). Although there was no significant difference in tumour size or T category between the two groups, colorectal NECs tended to more frequently be associated with lymphatic invasion (97% vs. 41%; *p* < 0.0001), vascular invasion (77% vs. 20%; *p* < 0.0001), lymph node metastasis (80% vs. 53%; *p* = 0.0001), and distant metastasis (37% vs. 14%; *p* = 0.0001), leading to a more advanced disease stage (stage III/IV, 83% vs. 55%; *p* = 0.0053) compared with those of 100 conventional ACs.

**Table 1:** Comparison of clinicopathologic features between NECs and ACs after matching tumour site.

	30 NECs	100 ACs	*p* value
	Number (%)	Number (%)	
Age (years), median (range)	67 (44–83)	62 (33–81)	0.07
**Sex**			
Male	20 (67)	57 (57)	0.4011
Female	10 (33)	43 (43)	
**Underlying disease**			
HNPCC	1 (3)	0	
FAP	1 (3)	0	
**Tumor location**			
Right-sided	9 (30)	30 (30)	
Left-sided	7 (23)	23 (23)	
Rectum	14 (47)	47 (47)	
Tumor size (cm), median (range)	5.65 (1.6–16)	5.4 (0.5–15)	0.1535
**Tumor differentiation**			
Low grade		89	
High grade	30	11	
**Histologic subtype**			
Small cell	12 (40)		
Large cell	18 (60)		
Mitoses/2 mm^2^, median (range)	62.5 (12–141)		
Ki-67 index(%), median (range)	75 (50–95)		
**Coexisting component**			
Adenocarcinoma	7 (23)		
Tubular adenoma	3 (10)		
**Depth of invasion**			
T1	1 (3)	6 (6)	0.1247
T2	4 (13)	12 (12)	
T3	19 (63)	76 (76)	
T4	6 (20)	6 (6)	
**Lymphatic invasion**			
No	1 (3)	59 (59)	< 0.0001
Yes	29 (97)	41 (41)	
**Vascular invasion**			
No	7 (23)	80 (80)	< 0.0001
Yes	23 (77)	20 (20)	
**N category**			
N0	6 (20)	47 (47)	0.0001
N1	6 (20)	33 (33)	
N2	18 (60)	20 (20)	
**M category**			
M0	9 (30)	91 (91)	0.0001
M1	11 (37)	14 (14)	
**Metastatic sites**			
Liver	6 (20)	7 (7)	> 0.9999
Others	5 (17)	7 (7)	
**Pathologic AJCC stage**			
I/II	5 (17)	45 (45)	0.0053
III/IV	25 (83)	55 (55)	

*HNPCC* hereditary nonpolyposis colorectal cancer, *FAP* familial adenomatous polyposis.

### Immunohistochemistry and microsatellite instability status

**Figure 1:**
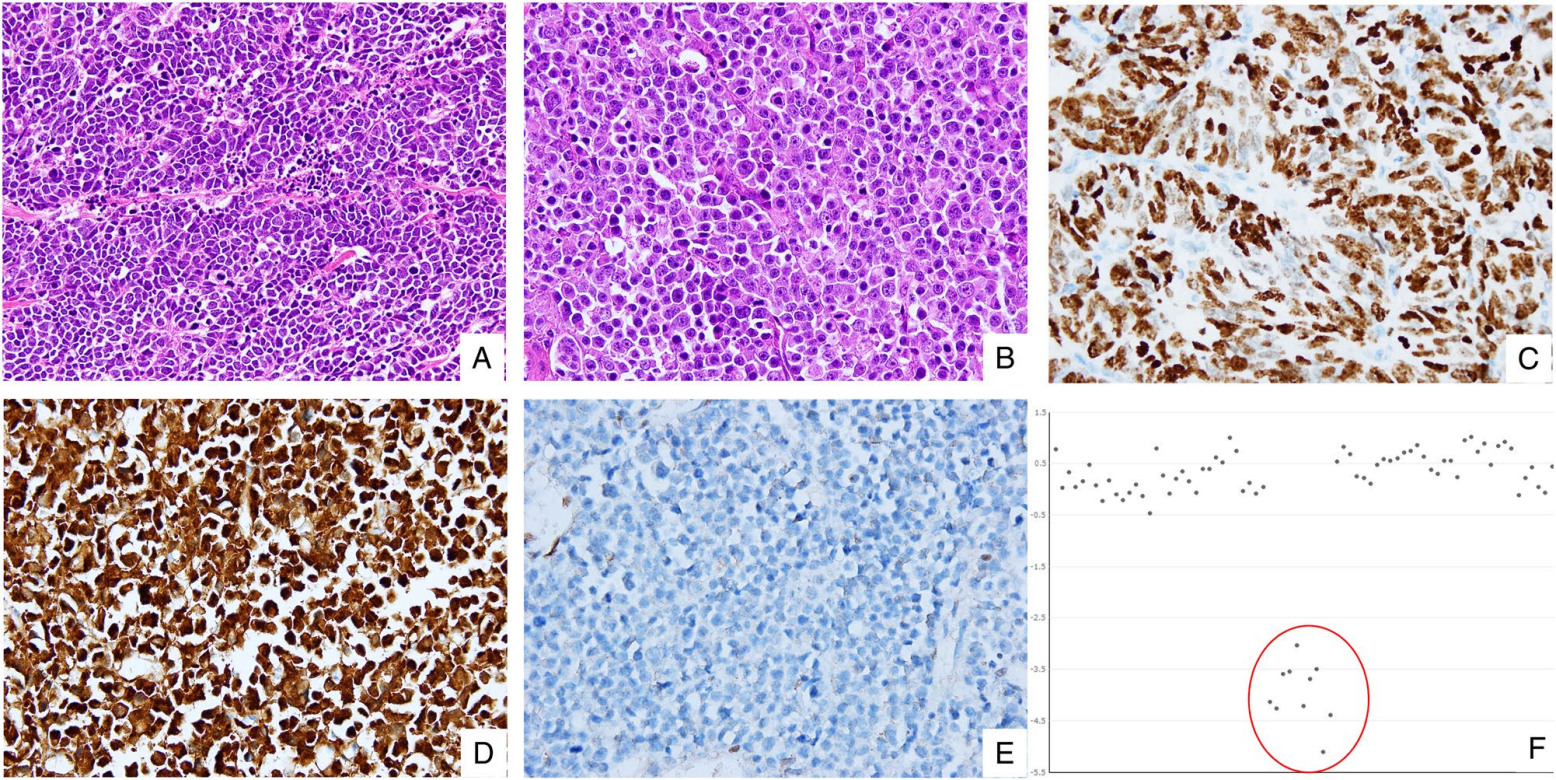
Rb1/p16 pathway deregulation in colorectal NECs revealed by immunohistochemistry and next-generation sequencing. (**A**) Representative micrograph of small-cell NEC displaying small to medium sized cells with a high nuclear-cytoplasmic ratio, scant cytoplasm and frequent mitotic figures. (**B**) Representative micrograph of large-cell NEC showing large polygonal cells with prominent nucleoli and moderate amount of eosinophilic cytoplasm. (**C**) Intense and diffuse nuclear staining for p53. (**D**) Intense and diffuse cytoplasmic staining for p16. (**E**) Complete loss of Rb1 expression. (**F**) Deep deletion of *RB1* on a normalized sequencing depth (red circle).

The immunohistochemistry and microsatellite instability results are provided in Table 2. Among 30 colorectal NECs, thirteen (43%) showed aberrant p53 protein expression, with overexpression or complete loss. Fourteen (47%) tumours displayed complete loss of Rb1 accompanied by overexpression of p16, as illustrated in Fig. 1. Three (10%) tumours expressed both Rb1 and p16. Among them, only one tumour showed diffuse strong positivity for both Rb1 and p16. Other two Rb1 positive tumours revealed patchy staining pattern (10% and 20% of cells) for p16. All p16 positive tumours were negative for HPV. There was one (3%) tumour with loss of MSH2 and MSH6 proteins, and the tumour was shown to have a high microsatellite instability status by subsequent microsatellite instability testing. Among 100 colorectal ACs, 35 (35%) showed aberrant expression of the p53 protein and no expression of Rb1. Three (3%) poorly differentiated ACs displayed strong expression of p16. There were seven (7%) tumours with loss of MLH1 and PMS2 proteins, and these tumours also had high microsatellite instability based on microsatellite instability testing.

**Table 2:** Comparison of immunohistochemistry and microsatellite instability status between NECs and ACs.

	NEC	AC	*p* value
Total number (%)	30 (100)	100 (100)	
**P53**			
Intact	17 (57)	65 (65)	0.518
Aberrant	13 (43)	35 (35)	
**RB1**			
Negative	14 (47)	100 (100)	
Positive	16 (53)	0	
**P16**			
Negative	13 (43)	97 (97)	< 0.0001
Positive	17 (57)	3 (3)	
**MMR proteins**			
Intact	29 (97)	93 (93)	0.6807
Loss	1 (3)	7 (7)	
**MSI**			
MSS	28 (93)	92 (92)	0.5155
MSI-L	1 (3)	1 (1)	
MSI-H	1 (3)	7 (7)	

*MMR* mismatch repair, *MSI* microsatelllite instability testing, *MSS* microsatellite stable, *MSI-L* microsatellite instability-low, *MSI-H* microsatellite instability-high.

### Molecular data

**Figure 2:**
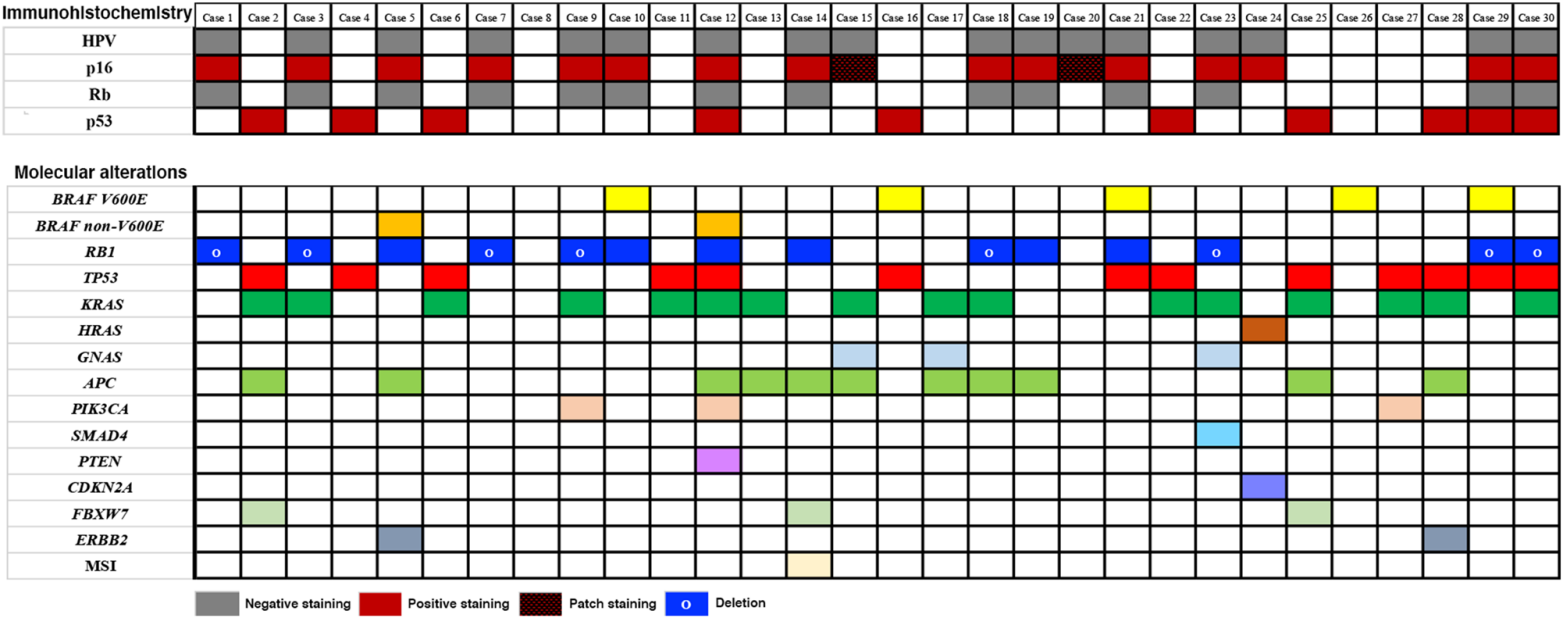
Heat map describing immunohistochemical results and somatic mutations identified in each case. Each row represents one case, and each row represents one gene. The above bar graph demonstrates the incidence of corresponding gene alterations.

All thirty NEC samples with more than 70% tumour cellularity were examined using the 382-gene panel. The somatic mutations and gene alterations identified in the 30 NEC tumours are detailed in Fig. 2. Activating *BRAF* mutations were detected in 7/30 (23%) tumours. The p.V600E *BRAF* mutations was observed in 5/7 (71%) tumours. Two (29%) tumours harboured *BRAF* mutations p.D594G and p.K601E. Activating *RAS* mutations (16 *KRAS* and 1 *HRAS* mutation) were identified in 16/30 (53%) tumours, predominantly in codons 12 and 13. *GNAS* mutations p.R201C and p.R201H were detected in three tumours (10%). *TP53* mutations were identified in 13/30 (43%) tumours but without consistently mutated hot spots. Fourteen (47%) NEC tumours showed distinct alterations in *RB1*, accompanied by loss of Rb1 and overexpression of p16 by immunohistochemistry. *RB1* alterations in 8/30 (27%) tumours presented predominantly as homozygous biallelic deletions, and activating *RB1* mutations were observed in 6 (20%) tumours. Moreover, alterations of *RB1* co-occurred with *TP53* mutations in 4/13 (31%) tumours and frequently occurred alone. Among three tumours expressed Rb1 and p16, one Rb1 positive tumour with diffuse labelling for p16 was found to have a CDKN2A mutation by NGS. However, two Rb1 positive tumours with patchy labelling for p16 did not show relevant molecular alterations with Rb1/p16 pathway. Additionally, mutations in various genes were occasionally detected, including *TET2, NOTCH1, NKX2-1, CHECK2, ATM, TS1, TS2, PTCH2, RHOA, DOT1L, ZNRF3,* and *ANTXR2*. The majority of these mutations are of unknown functional consequence.

**Figure 3:**
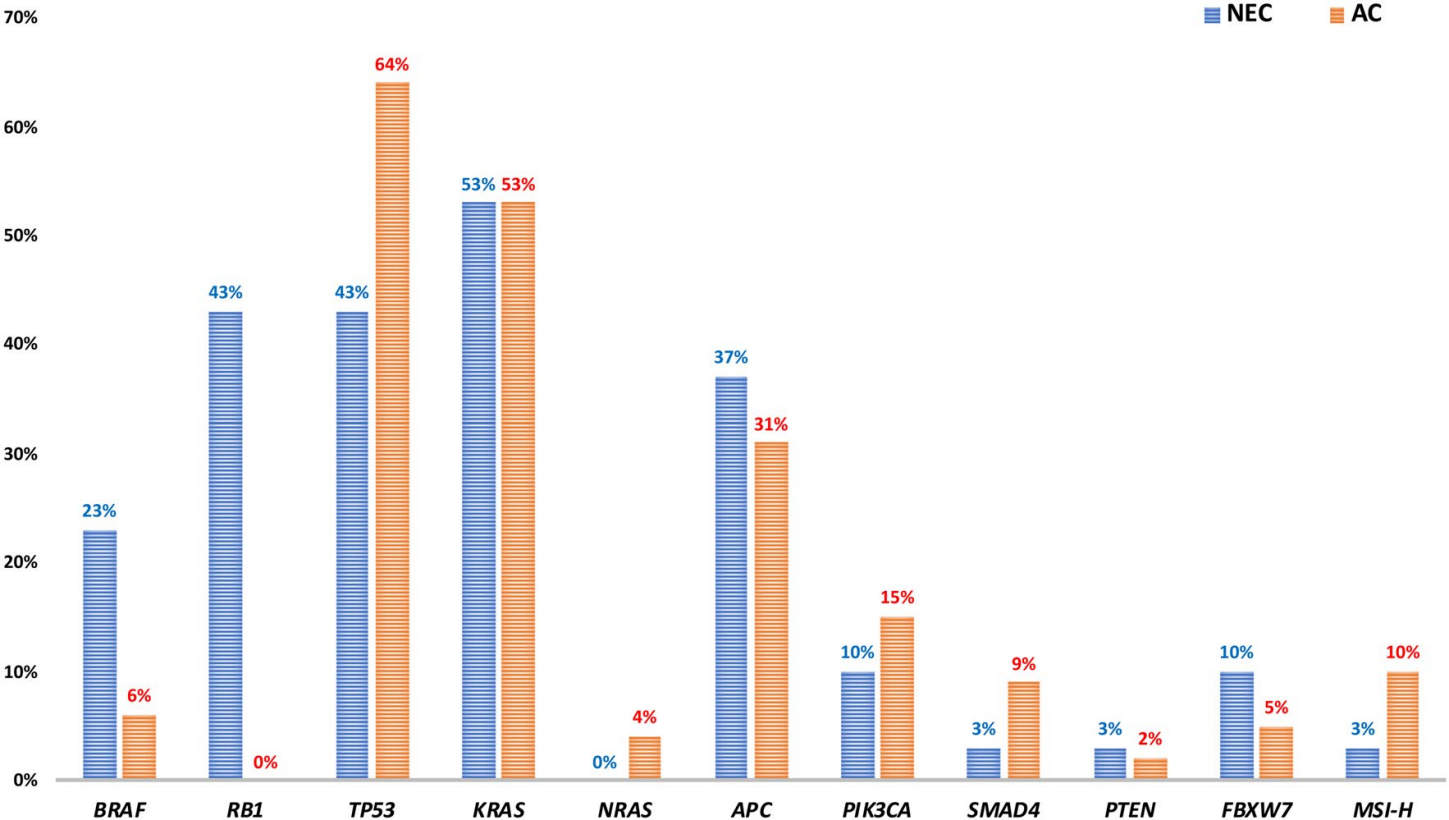
Bar graph depicting frequencies of somatic mutations in colorectal neuroendocrine carcinomas (NECs) and adenocarcinomas (ACs) after matching tumour site. There is a significant difference in frequencies of *BRAF* mutations between two groups (*p* = 0.0112).

Molecular data for one hundred colorectal ACs matched by tumour site were analysed for comparison. The results of the comparative analysis of gene mutations in NECs and ACs are depicted in Fig. 3. Compared with the 100 AC group samples, the NEC samples frequently harboured *BRAF* mutations (23% vs. 6%; *p* = 0.0112), particularly p.V600E (71%). The frequencies of identified *KRAS* mutations were similar in both groups (53% (NEC) vs. 53% (AC); *p* = 1.0). Although the frequencies of *TP53* and *APC* mutations in the NEC group were slightly higher than those in the AC group (43% vs. 35%; p = 0.0546, 37% vs. 31%; *p* = 0.6569, respectively), *PIK3CA* mutations were less frequently identified in the NEC group (10% vs. 15%; *p* = 0.7633). Overall, there was no statistically significant difference in the frequencies of mutations in *TP53, APC*, or other genes, including *SMAD4*, *PIK3CA*, *PTEN*, and *FBXW7*, which have been reported to be mutated in colorectal ACs by next-generation sequencing.

### Clinical outcome

**Figure 4:**
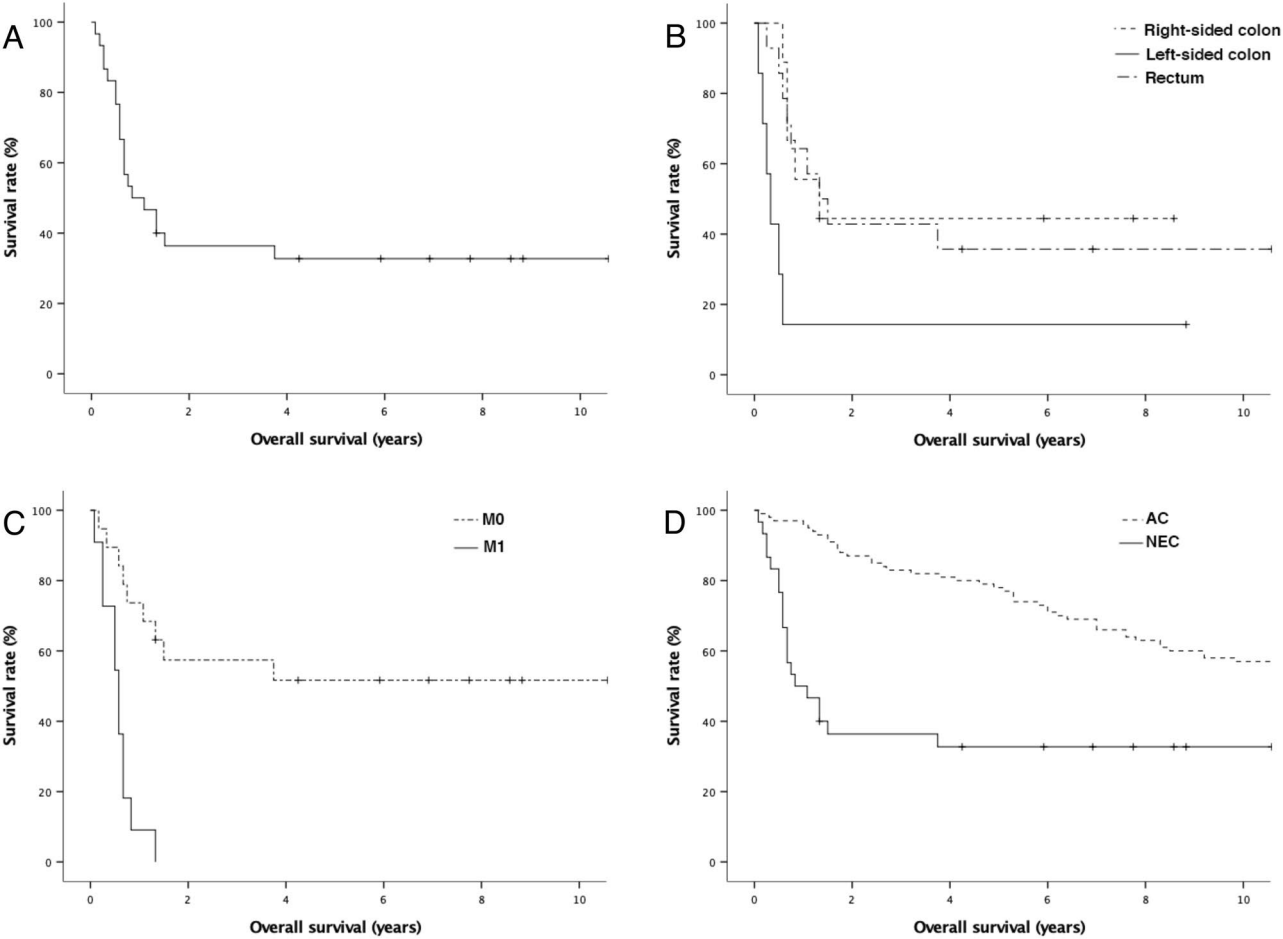
Kaplan–Meier cumulative survival curves. (**A**) Overall survival. (**B**,**C,D**) Survivals according to the tumour sites; right-sided colon vs. left-sided colon vs. rectum (*p* = 0.013); M0 vs. M1 (*p* < 0.001); AC vs. NEC (*p* < 0.001).

Follow-up data were available for all 30 patients with colorectal NEC and 100 patients with AC. At the time of the last follow-up, 21 (70%) patients had expired due to colorectal NEC, and 9 (30%) patients were alive with no evidence of recurrence. The 1-, 3-, and 5-year overall survival (OS) rates for all 30 patients were 46.7%, 36.4%, and 32.7%, respectively, with a median follow-up of 11.5 months (range 1 to 132 months). Kaplan–Meier survival analysis showed a high T category (*p* = 0.031), lymph node metastasis (p = 0.02), and distant metastasis at diagnosis (M category) (*p* < 0.001) to be significantly associated with shorter OS (Fig. 4). In addition, patients with NEC in the left colon showed an inferior OS than patients with NEC in the rectum or the right colon (*p* = 0.014). In multivariate analysis, distant metastasis at diagnosis (M category) was the only independent prognostic factor related to OS (Table 3). Regarding other potential molecular alterations related to survival, there were no significant differences in OS rates between groups with mutated and wild-type *BRAF*, groups with mutated and wild-type *RB1*, or groups with mutated and wild-type *TP53*.

**Table 3:** Univariate and multivariate analysis for overall survival.

Variable	Univariate analysis		Multivariate analysis	
	Hazard ratio (95% CI)	*p* value	Hazard ratio (95% CI)	*p* value
T category (T1 vs. T2 vs. T3 vs. T4)	2.2 (1.01–4.793)	0.041	1.709 (0.71–4.115)	0.232
N category (N0 vs. N1 vs. N2)	2.46 (1.176–5.127)	0.006	1.951 (0.89–4.278)	0.105
M category (M0 vs. M1)	6.073 (2.221–16.603)	< 0.001	4.278 (1.507–12.146)	0.006
*BRA*F mutations (mutant vs. wild type)	1.391 (0.5–3.873)	0.528		
*TP53* mutations (mutant vs. wild type)	0.596 (0.234–1.518)	0.278		
*RB1* alterations (alteration vs. wild type)	1.898 (0.784–4.594)	0.155		

*CI* confidence interval.

We also compared the OS rates of patients with colorectal NEC and those with 100 colorectal adenocarcinomas (Fig. 4). Among 100 patients with colorectal adenocarcinoma, 54 (54%) patients died of the disease. The median overall survival was 164.5 months, with 1-, 3-, and 5-year survival rates of 96%, 83%, 78%, respectively. Overall, the OS rate of patients with colorectal NEC was poorer than that of patients with colorectal adenocarcinoma (*p* < 0.001).

## Discussion

Poorly differentiated colorectal NEC is a rare malignant neoplasm accounted for only 0.6% of all CRCs^1^. The clinicopathologic features and prognosis of colorectal NECs have been described in a few relatively large series. With a median OS of 11.5 months, the findings demonstrate that poorly differentiated colorectal NEC has an aggressive biologic behaviour with a poor clinical outcome, in line with prior studies. A retrospective study of 100 colorectal NECs analysed at M.D. Anderson Cancer Center reported a median age at diagnosis of 55 years (range 33–88 years), with 51% of the patients being men^23^. The majority of poorly differentiated NECs had a small-cell morphology (89%) rather than a large-cell morphology (8%), and metastatic disease was noted in 64 (64%) patients at diagnosis. In another retrospective analysis of data from the National Cancer Database (2004–2015) consisting of 1208 poorly differentiated colorectal NECs, the median age at diagnosis was 65 years, and 50% of patients were male^3^. A small-cell morphology was slightly more frequent than a large-cell morphology (54% vs. 46%). Lymphovascular invasion was observed in 25% of NEC cases, and approximately 55% of patients had advanced disease (III/IV) at diagnosis. In addition, Takaziwa et al. reported that patients with colorectal NECs were diagnosed at older age (median, 68 years) and with a male predominance (2:1)^7^. Among 25 colorectal NECs, a large-cell morphology was more commonly observed than was a small-cell type (64% vs. 36%). NEC tumours showed high rates of lymph node metastasis (82%) and distant metastasis (44%) and were at a more advanced stage (stage III/IV, 92%). In the present series, the patients were diagnosed at an older age (mean age: 67 years), with a male predominance (male to female ratio: 2:1), and among the 30 NEC tumours, a large-cell morphology was more frequently observed than a small-cell morphology (60% vs. 40%). Compared with typical ACs, NECs revealed multiple adverse prognostic pathologic factors, including frequent lymphatic invasion (97%), vascular invasion (77%), lymph node metastasis (80%), and distant metastasis at diagnosis (70%), which are associated with advanced TNM stage (stage III/IV, 83%). Interestingly, based on the data from the Japanese and current series, patients with colorectal NECs in East Asian countries tend to have more adverse clinicopathologic factors related to advanced TNM staging than do patients in Western countries. However, due to the rarity of this disease entity, discrepancy in the analysed clinicopathologic findings of colorectal NECs depending on racial/geographic differences or observer variations is unclear.

Unlike colorectal ACs, surgery alone is rarely curative for poorly differentiated NECs, and the survival benefit from surgery is controversial in prior studies^24,25^. According to poorly differentiated colorectal NEC data from the National Cancer Database (2004–2015), the median OS for patients who underwent surgical resection was 10.5 months compared with 6.9 months for patients who did not undergo surgery (p < 0.001)^3^. In our series, the median OS was 11.5 months; the 3-year survival was 36.4%, and the 5-year survival was 32.7%. The patients in our series with surgically resected colorectal NECs had a better OS than those who did not undergo surgery in the data from the National Cancer Database (11.5 months vs. 6.9 months). Regardless, it is uncertain whether this correlation is due to selection bias or true therapeutic effects. Thus, further studies about the survival benefit of surgical resection in treating colorectal NECs are needed.

Approximately 8% of CRCs harbour *BRAF* mutations, predominantly in exon 15, namely, p.V600E^26^. *BRAF* mutation is known to be prevalent in tumours of the proximal colon and tumours with poor differentiation (grade 3 or 4), mucinous histology, and high microsatellite instability^26^. However, the frequencies of *BRAF* mutations in poorly differentiated colorectal NECs are controversial (Table 4). Klempner et al. reported that ten (9%) of one hundred colorectal NECs harboured *BRAF* mutations in the form of p.V600E (80%), p.G469A (10%), or p.R671Q (10%)^27^. Olevian et al. found that seventeen (59%) of twenty-nine NECs had *BRAF* mutations in the form of p.V600E (88%), p.F595L (6%), or p.K601N (6%) by Sanger sequencing^8^. Additionally, Jesinghaus et al. reported different frequencies of identified *BRAF* mutations between MANECs and pure NECs, with an average of 33.3% of samples showing *BRAF* mutations^9^. In their study, colorectal MANECs more frequently had *BRAF* mutations (37%, 7/19) than pure NECs (25%, 2/8). In our series, seven (23%) of thirty pure NECs harboured *BRAF* mutations in the form of p.V600E (71%), p.D694G (14%), or p.K601E (14%). For comparison, six (6%) of a hundred conventional colorectal ACs harboured *BRAF* mutations in the form of p.V600E (83%) or p.G466V (17%). Because different frequencies of *BRAF* mutations in CRC have been reported depending on tumour location, we extracted the molecular data of conventional colorectal ACs matched by tumour location with the colorectal NEC group. Based on our data, *BRAF* mutations were more frequently identified in poorly differentiated colorectal NECs than in conventional colorectal ACs (23% vs. 6%, *p* = 0.0112). Several case series of *BRAF*-targeted therapy have shown promising responses in patients with *BRAF*-mutated CRCs, including poorly differentiated NECs^27,28^. Considering that colorectal NECs harbour more *BRAF* mutations than do other types of CRC, it is critical to perform molecular testing for *BRAF* in patients with colorectal NEC to optimize patient selection for promising therapeutic options, such as BRAF inhibitors.

**Table 4:** Comparison of frequently identified mutated oncogenic genes in previous literatures and current study.

Series	Country	Year	Case number	Methodology	*RB1*	*BRAF*	*TP53*	*KRAS*	*APC*	*PIK3CA*	*PTEN*	*FBXW7*	MSI-H
Lee and Sung	South Korea	2020	30 NECs	382 gene	14 (47%)	7 (23%)	13 (43%)	16 (53%)	11 (37%)	3 (10%)	1 (3%)	3 (10%)	1 (3%)
Shamir et al.	United State	2019	24 NECs	479 gene	14 (58%)	1 (4%)	12 (50%)	7 (29%)	12 (50%)	3 (13%)	5 (21%)	0	2 (8%)
Jesinghaus et al.	Germany	2017	19 MANECs and 8 NECs	32 gene	1 (4%)	9 (33%)	13 (48%)	6 (22%)	8 (30%)	1 (4%)	3 (11%)	3 (11%)	2 (7%)
Woischke et al.	Germany	2017	10 MANECs and 5 NECs	50 gene	5%	3%	13%	11%	10%	5%	1%	3%	
Olevian et al.	United State	2015	29 NECs	Sanger		17 (59%)		5 (17%)					2 (7%)
Takizawa et al.	Japan	2015	24 NECs	Sanger		1 (4%)	5 (21%)	2 (8%)	1 (4%)				

*Sanger* Sanger sequencing.

*RB1* is a critical negative regulator of the Rb1/p16 pathway, which controls the G1 checkpoint of the cell cycle^29^. Inactivating mutations or homozygous deletions in *RB1* have been reported in neuroendocrine neoplasms of the lung, gastrointestinal tract, and prostate^11,30–32^. Furthermore, a comprehensive whole-genome sequencing study of 110 pulmonary small-cell carcinomas found near-universal biallelic inactivation of *RB1* via mutations, deletions, and complex rearrangements^31^. Nonetheless, there are contradicting data on the frequency of molecular alterations in *RB1* in colorectal NECs. Only one (3.7%) patient with heterozygous *RB1* loss was reported in a previous molecular analysis of 27 colorectal MANECs and NECs by targeted next-generation sequencing^9^. In contrast, a recent molecular analysis of 24 colorectal NECs and MANECs revealed that fourteen (58%) tumours showed biallelic alterations in *RB1* with next-generation sequencing using a 479-gene panel^11^. Similarly, in our series, fourteen (47%) pure NECs displayed genomic alterations in *RB1*, including both mutations and deep deletions, with the latter being more prevalent (57%) than the former (43%). The tumours with *RB1* alterations also exhibited aberrant expression of Rb1 and p16 by immunohistochemistry. Genomic alterations in *RB1* accompanied by loss of Rb1 and overexpression of p16, representing deregulation of the Rb1-p16 pathway, are commonly observed in colorectal NECs. Prior and our data support that deregulation of the Rb1-p16 pathway, as confirmed by immunohistochemistry and next-generation sequencing, plays a critical role in the histogenesis of colorectal NECs, as it does in small-cell lung cancer. Unlike a study result by Shamir et al., we failed to find an association between p16 positive colorectal NECs and HPV infection. The difference of HPV infection status in colorectal NEC tumours between two studies may be caused by selection bias, presence of NEC tumours coinfected with HPV, or others. Further studies would be needed for investigating the role of HPV infection in the histogenesis of a subset of colorectal NEC tumours.

The frequencies of other oncogenic driver genes commonly identified in CRC, such as *TP53, KRAS, APC, PIK3CA,* and *PTEN*, have been inconsistently reported in prior studies (Table 4)^7–11^. Two recent studies demonstrated relatively high mutation rates of *TP53, KRAS,* and *PIK3CA*, roughly similar to those of our molecular data^9,11^. However, two other studies showed relatively lower mutation rates for these genes^8,10^. These studies did not compare these mutated genes of colorectal NECs with those of colorectal ACs, and it remains unclear whether there are significant differences in these mutated genes in colorectal NECs. Thus, the present study compared these mutations of colorectal NECs with those of colorectal ACs after tumour site matching and detected no significant differences in the frequencies of *TP53*, *KRAS*, *APC*, *PIK3CA*, *PTEN*, *GNAS*, and *SMAD4* mutations between 30 NEC tumours and 100 AC tumours. The almost parallel frequencies and distributions of the main oncogenic gene mutations, except for *BRAF*, identified between the NEC and AC groups indicate that colorectal NECs are genetically similar to colorectal ACs, suggesting that colorectal NECs arise from the same origin as colorectal ACs, with intestinal glands likely serving as the primary origin.

In summary, our study demonstrates that colorectal NECs display an aggressive biologic behaviour with multiple adverse clinicopathologic prognostic factors and a poor clinical outcome. In molecular analysis, *BRAF* mutations, predominantly p.V600E, were more frequently identified in poorly differentiated NECs than in conventional ACs. Additionally, almost exclusive deregulation of the Rb1/p16 pathway was revealed by immunohistochemistry and next-generation sequencing. Frequencies and distributions of the main mutated oncogenic driver genes, except *BRAF*, and the microsatellite instability status were similar in NECs and ACs. These findings provide beneficial information for the use of potential therapeutics such as BRAF inhibitors and a better understanding of the histogenesis of this tumour.
